# Differentiation of pluripotent stem cells into striatal projection neurons: a pure MSN fate may not be sufficient

**DOI:** 10.3389/fncel.2014.00398

**Published:** 2014-12-02

**Authors:** Amy E. Reddington, Anne E. Rosser, Stephen B. Dunnett

**Affiliations:** ^1^The Brain Repair Group, School of Biosciences, Cardiff UniversityCardiff, UK; ^2^Department of Psychological Medicine and Neurology, Cardiff UniversityCardiff, UK

**Keywords:** neuronal transplantation, pluripotent stem cell grafts, embryonic stem cells, iPS cells, striatal grafts, striatal fate, medium spiny neurons, Huntington’s disease

## Abstract

Huntington’s disease (HD) is an autosomal dominant inherited disorder leading to the loss *inter alia* of DARPP-32 positive medium spiny projection neurons (“MSNs”) in the striatum. There is no known cure for HD but the relative specificity of cell loss early in the disease has made cell replacement by neural transplantation an attractive therapeutic possibility. Transplantation of human fetal striatal precursor cells has shown “proof-of-principle” in clinical trials; however, the practical and ethical difficulties associated with sourcing fetal tissues have stimulated the need to identify alternative source(s) of donor cells that are more readily available and more suitable for standardization. We now have available the first generation of protocols to generate DARPP-32 positive MSN-like neurons from pluripotent stem cells and these have been successfully grafted into animal models of HD. However, whether these grafts can provide stable functional recovery to the level that can regularly be achieved with primary fetal striatal grafts remains to be demonstrated. Of particular concern, primary fetal striatal grafts are not homogenous; they contain not only the MSN subpopulation of striatal projection neurons but also include all the different cell types that make up the mature striatum, such as the multiple populations of striatal interneurons and striatal glia, and which certainly contribute to normal striatal function. By contrast, present protocols for pluripotent stem cell differentiation are almost entirely targeted at specifying just neurons of an MSN lineage. So far, evidence for the functionality and integration of stem-cell derived grafts is correspondingly limited. Indeed, consideration of the features of full striatal reconstruction that is achieved with primary fetal striatal grafts suggests that optimal success of the next generations of stem cell-derived replacement therapy in HD will require that graft protocols be developed to allow inclusion of multiple striatal cell types, such as interneurons and/or glia. Almost certainly, therefore, more sophisticated differentiation protocols will be necessary, over and above replacement of a specific population of MSNs. A rational solution to this technical challenge requires that we re-address the underlying question—what constitutes a functional striatal graft?

## Introduction

In humans, the adult striatum is composed of two histologically equivalent nuclei—the caudate nucleus and the putamen—which together, with other core nuclei in the depths of the forebrain, make up the basal ganglia (Jain et al., 2001). The striatum is connected through independent and parallel pathways with widespread areas of the neocortex, the pallidum, the thalamus and the brainstem and plays a vital role in the co-ordination of movement (primary motor control), emotions, and cognition (Jain et al., 2001). In Huntington’s disease (HD), a genetic mutation in the huntingtin (*htt*) gene results in early loss of the medium sized striatal projection neurons (MSNs) of the striatum, atrophy of striatal volume (Vonsattel et al., 1985), and disruption of functional communication through the basal ganglia pathways, leading to motor, cognitive and psychiatric decline.

Currently, there is no known cure for HD. However, the specificity of cell loss seen at least in early stages of the disease—principally involving loss of the MSN projection neurons—has made cell transplantation a viable therapeutic prospect (Dunnett and Rosser, 2014). Transplants using primary human fetal striatal tissue have demonstrated “proof-of-principle” that cell replacement is feasible, that the grafts are safe and do not accelerate disease progression (Rosser et al., 2002), and importantly have significant, although incomplete, functional recovery in at least some patients (Bachoud-Lévi et al., 2000, 2006; Barker et al., 2013). However, due to the ethical issues associated with the use of human fetal cells (PFCs) obtained from elective termination of pregnancies, the logistical issues arising from the amount of fetal tissue required per patient, and the difficulties in achieving an appropriate level of standardization and quality control for tissues derived from such a recurrent clinical source, better renewable sources of cells for transplantation are under active exploration. Human pluripotent stem cells (hPSCs) are the leading contender under consideration, by virtue of their capacity for indefinite expansion as well as their potential for differentiation to essentially any mature fate; the principle sources being embryonic stem cells (ESCs) and/or induced pluripotent stem cells (iPSCs), followed by directed differentiation *in vitro* towards a specific neural phenotype (or phenotypes) prior to transplantation as required for each disease target.

Over the last 5 years there has been some considerable success in producing MSN-like neurons from PSC sources, including from hESCs that have been directed to a neuronal phenotype, and then ventralised using sonic hedgehog or its agonist, purmorphamine (El-Akabawy et al., 2011). To date a small number of published protocols have also reported differentiation of MSN-like cells *in vitro* and post transplantation in the rodent brain, with limited evidence that the cells could integrate into the host neural circuitry and receive dopaminergic inputs from the midbrain and glutamatergic inputs from the cortex while projecting fibers to the substantia nigra (Aubry et al., 2008; Ma et al., 2012; Delli Carri et al., 2013; Arber et al., in press). These protocols and key findings are summarized in Table 1. However, although there is some evidence that the transplanted cells corrected motor deficits in a rodent model of striatal neurodegeneration, in no case to date have the cells demonstrated a full repertoire of functional improvements that would reliably indicate that they are indeed authentic MSNs. Thus, we must ask whether generating neurons with the principal characteristic marker of the MSN phenotype, *viz*. that they express the dopamine and cyclic AMP-regulated neuronal phosphoprotein, DARPP-32, is a sufficient (or indeed necessary) condition for optimizing functional recovery. Are other features of the intact striatum or other neuronal subtypes equally important for an optimal, functional graft?

**Table 1:** **Studies of grafts of PSCs into rodent striatum**.

Protocol	Cell source	Host treatment	Transplant	Brief summary of results
Dinsmore et al. (1996)	mESC (E14TG2a; D3)	QA lesioned rat striatum Daily cyclosporine	100,000–1,000,000 cells. Survival up to 6 wk	Treatment of pluripotent ES cell cultures with retinoic acid (RA) induced populations of GABAergic expressing neurons (no specific neuronal type was targeted). Grafts containing 100,000 cells produced biggest grafts. Grafts stained positively for AChE, Thy1.2, TUJ1, NSE and GABA. No neurite outgrowth was determined due to the absence of a species specific marker.
Kallur et al. (2006)	NSCs from primary striatal tissue expanded *in vitro* as neurospheres	Intact striatum of neonatal rat (2–3 days) No immune suppression	100,000 cells Survival 4 and 16 wk	At 4 months 6–10% of cells had survived, the majority were located in the striatum but some had migrated to the GP, cortex or corpus callosum. At 4 months, the number of NESTIN positive cells had decreased whereas the number of DCX and NEUN cells had increased compared to at 1 month. Some cells were GFAP positive and the majority of all neurons stained positively for parvalbumin. A selection of neurons had morphology characteristic of mature neurons with long branching processes and visible dendritic spines whereas others had more astrocyte/oligodendrocyte like morphology.
Joannides et al. (2007a)	H9; HUES9	QA lesioned rat striatum Daily cyclosporine	100,000–250,000 cells Survival at 6 wk	Cells grown under optimized and fully defined human neuralizing medium under substrate-free conditions. No tumors evident following transplantation. Doublecortin (DCX) and NeuN positive neurons identified. Limited GFAP staining also evident- suggestive of some astrocyte differentiation. No DARPP-32 present, no sign of neuron migration from graft core.
Song et al. (2007)	Miz-hESC1	QA lesioned rat striatum Daily cyclosporine	20,000 cells Survival at 3 wk	Some cells migrated to the cortex and formed “aggregates” that were Nestin positive/NeuN negative. Some cells were GFAP positive. Cells remaining in the striatum migrated to the lesion core and were DCX and GAD67 positive/DARPP-32 negative. Improved apomorphine rotations at 1, 2 and 3 weeks compared to sham group. No overgrowth reported.
Aubry et al. (2008)	SA-01 (H9)	QA lesioned striatum in nude rat No immune suppression	50,000–200,000 cells Survival 4–6 wk	Grafts from “early” stage cells (day 21–30 of the protocol) showed no DARPP-32 expressing cells and developed “teratoma-like regions” whilst cells grafted from the “later” stage (day 46–59) of the protocol showed clusters of DARPP-32 (21% of NeuN positive neurons)/AChE negative cells and contained P-zones. The cells had medium sized bodies (10–16 µm), were bi-polar and showed extensive neurite outgrowth. There was overgrowth 13–15 weeks after the graft. No functional assessment.
Lee et al. (2009)	Adipose-derived stem cells (ASCs)	QA lesioned rat striatum Daily cyclosporine	100,000 cells	Grafts reduced apomorphine-induced rotations (1–4 weeks after), lesion volume, and striatal apoptosis.
		60 day old R6/2 mouse Daily cyclosporine	500,000 cells	Grafts improved rotarod performance and limb clasping, increased survival, attenuated the loss of striatal neurons, and reduced the Htt+ aggregates. Cells expressed DCX, TUJ1 and GAD.
Nasonkin et al. (2009)	hESCs (BG01)	Unlesioned Striatum in nude rats No immune suppression	15,000 cells Survival 1.5, 3 and 6 mo.	Following transplantation nestin and DCX expression decreased and TUJ1 increased. DARPP-32, calretinin and parvalbumin expression at 6 months, no GAD67. Synaptophysin evident and sparse Glur2/3. Axonal projections to the GPe and sub thalamic nucleus seen. No overgrowth, no functional assessment.
Vazey et al. (2010)	ENVY (GFP-expressing)	QA lesioned rat striatum Daily cyclosporine	75,000 cells Survival 4 and 8 wk	Grafted cells expressed MAP2 and NeuN at both time points, no DARPP-32 or GAD67. Overgrowth seen in 1 graft at 8 weeks. No functional assessment.
Ma et al. (2012)	hESCs	QA lesioned striatum in SCID mice No immune suppression	100,000 cells Survival 16 wk	Shorter protocol than previous attempts to generate LGE neural precursors that predominately differentiated into DARPP-32-expressing neurons. Cells were grafted after 40 days *in vitro* and 4 months after transplantation showed no over-growth and were positive for DARPP-32, MEIS2, CTIP2, enkephalin and substance P. Cells were multipolar, branched and had numerous dendritic buttons revealed through synaptophysin staining. Small populations of the neurons also expressed ChAT, vGLUT1, 5-HT, TH and calbindin. Functional recovery was seen on the rotarod and through an increase in stride length which was attributed to the host cortical and nigral inputs to the grafts as well as the projections afforded to the SN from the grafts.
El-Akabawy et al. (2012)	cmyc-ER^TAM^ hNSC (STROC05)	R6/2 HD mouse No immune suppression	75,000 cells Survival up to 6 wk	Tested on a battery of behavior tests including rotarod, Cells did not diminish disease progression, possibly due to the short life span of the mouse (16 weeks). There was no DARPP-32. There was no sign of graft rejection but this does not rule out an early immune response on the graft.
Delli Carri et al. (2013)	hESCs (H9 and HS401)	QA lesioned rat striatum Daily cyclosporine	500,00 cells Survival at 3, 6 and 9 wk	Used the same concentration of SHH as Ma et al., to induce a ventral telencephalic identity and characterized extensively to ensure LGE precursors. Cells grafted at Day 38 of the protocol. At 6 and 9 weeks MAP2ab mad TUJ1 positive neurons were seen. At 9 weeks post-transplant FOXP1, FOXP2 and DARPP-32 staining was found in the grafts but not quantified. Projection of Nestin fibers into the intact striatum showed integration between host and graft. Amphetamine-induced rotations were compared before and after grafting from 3 weeks and results hinted at functional recovery, however animal numbers were too low to suggest a significant behavioral effect.
Nicoleau et al. (2013)	hESCs (H9)	QA lesioned striatum of nude rats. No immune suppression	100,000 cells Survival at 5 mo.	Optimized concentration of SHH and WNT signaling to produce human ventral telencephalic precursors that were characterized extensively before grating. Day 25 differentiated hESC grafted, DARPP-32 and FOXP1 found in grafts, as yet no behavioral assessment has been carried out.
Arber et al. (in press)	hESCs (H7) Activin protocol	QA lesioned rat striatum Daily cyclosporine	500,000 cells Survival at 4–16 wk.	DARPP-32 shown in grafts at 16 weeks containing CTIP2, FOXP2 and calbindin positive neurons. No overgrowth.

In order to address this question, it is instructive to revisit first the separate issue of what have we learned about the composition and organization provided by experimental grafts of “striatal” PFCs derived from the embryonic ganglionic eminence, for which we have considerable evidence of robust and reproducible functional recovery in both rodent and primate animal models. A rich background literature on the structural, neurochemical, morphological, connections, electrophysiology and behavioral reconstruction provided by PFC grafts, and their correspondence to normal striatum, is summarized in Table 2. Striatal PFC grafts, whether derived from human, primate or rat embryos, do not exclusively contain MSNs; they contain cells from the entire developing striatum (Bachoud-Lévi et al., 2000, 2006; Rosser et al., 2002; Barker et al., 2013) and have shown more convincing functional recovery and integration than so far seen from PSC-derived grafts, strongly suggesting that inclusion of other cell types of the intact striatum, such as interneurons, should be considered. This review will therefore attempt to give an overview of what we have learnt about repairing the intact striatum using PFC-derived cells (Table 2), and the implications for developing alternative PSC-based protocols for cell transplantation in HD.

**Table 2:** **Molecular, Anatomical and Functional features of intact striatum, primary fetal striatal grafts, and pluripotent stem cell-derived grafts ^†^**.

	Striatal grafts (selected refs)	Notes^*^	PSC grafts (all refs ^††^)
**Cell morphology (Golgi):**
MSN : 90–95% medium sized spiny projection	Roberts and DiFiglia (1988), Wictorin et al. (1989), Helm et al. (1990), Clarke et al. (1994), Olsson et al. (1995)	+ *[in P zones]*	?
SIN : 4–8% medium sized spiny and non-spiny	Roberts and DiFiglia (1988), Helm et al. (1990), Clarke et al. (1994)	+ *[in P zones]*	?
GCN : 1–2% giant aspiny neurons	Helm et al. (1990, 1992)	+ *[in P zones]*	?
Astrocytes : structural and reactive	Petit et al. (2002), Zhu et al. (2013)	+ *[patchy]*	+ Kallur et al. (2006), Joannides et al. (2007b), Song et al. (2007)
Oligodendrocytes : myelinating internal capsule		?	?
*[Microglia : in response to* *damage or inflammation]*	Freeman et al. (2000)	– *[throughout]*	?
*[pallidal-like, medium, aspiny]*	Graybiel et al. (1989), Clarke et al. (1994)	+ *[in NP zones]*	?
*[cortical-like, pyramidal—in NP* *zones]*	Clarke et al. (1994)	+ *[in NP zones]*	?
**Neurotransmitters**:	
MSN and SIN: GABA, GAD	Isacson et al. (1985), Roberts and DiFiglia (1988), Clarke and Dunnett (1993), Piña et al. (1994b)	+	−Nasonkin et al. (2009), Vazey et al. (2010)
			+ Dinsmore et al. (1996), Lee et al. (2006), Song et al. (2007), Arber et al. (in press)
MSN: Enk, proenkephalin	Roberts and DiFiglia (1988), Graybiel et al. (1989), Sirinathsinghji et al. (1990), Emerich et al. (1991), Campbell and Björklund (1995), Freeman et al. (2000)	+ *[in P zones]*	Ma et al. (2012)
MSN: Dyn, prodynorphin	Sirinathsinghji et al. (1990)	+	?
MSN: SP, preprotachykinin	Sirinathsinghji et al. (1990), Helm et al. (1992), Campbell and Björklund (1995), Freeman et al. (2000)	+ *[in P zones]*	Ma et al. (2012)
SIN: parvalbumin, PV	Capetian et al. (2009)	+	+ Saporta et al. (2001), Kallur et al. (2006), Nasonkin et al. (2009)
SIN: calretinin, CR	Freeman et al. (2000), Keene et al. (2007), Capetian et al. (2009)	+ *[in P zones]*	+ Saporta et al. (2001), Kallur et al. (2006), Nasonkin et al. (2009), El-Akabawy et al. (2011)
SIN: neuropeptide Y, preproNPY	Morris et al. (1989), Sirinathsinghji et al. (1990)	+ *[in P zones]*	?
SIN: somatostatin, SOM	Graybiel et al. (1989), Morris et al. (1989), Freeman et al. (2000), Capetian et al. (2009)	+ *[in P zones]*	?
SIN: nitric oxide synthase, NOS		?	?
GCN: Acetylcholine, ChAT, AChE	Graybiel et al. (1989), Helm et al. (1992), Freeman et al. (2000)	+ *[in P zones]*	+ Dinsmore et al. (1996), Ma et al. (2012)
calbindin, calbindin D28k	Graybiel et al. (1989), Freeman et al. (2000)	+ *[P and NP zones]*	+ Saporta et al. (2001), El-Akabawy et al. (2011), Ma et al. (2012), Arber et al. (in press)
**Molecular markers**
Pluripotent cell markers (Sox1 etc)		?	?
Neuronal precursors (TUJ1, Nestin, NSE, DCx etc)		?	+ Dinsmore et al. (1996), Kallur et al. (2006), Lee et al. (2006), Joannides et al. (2007b), Song et al. (2007), Nasonkin et al. (2009), Delli Carri et al. (2013)
zones of tumor/teratoma overgrowth		+/−	+ Song et al. (2007), Aubry et al. (2008), Vazey et al. (2010)
MSNs: DARPP-32	Wictorin et al. (1989), Labandeira-García et al. (1991), Campbell and Björklund (1995), Freeman et al. (2000), Keene et al. (2007)	+ *[in P zones]*	–Vazey et al. (2010), El-Akabawy et al. (2012) + (Kallur et al. (2006), Nasonkin et al. (2009), El-Akabawy et al. (2011), Ma et al. (2012), Delli Carri et al. (2013), Nicoleau et al. (2013), Arber et al. (in press)
MSNs: FoxP1, FoxP2, Ctip2		+/?	+ Ma et al. (2012), Delli Carri et al. (2013), Nicoleau et al. (2013), Arber et al. (in press)
SINs: NADPH diaphorase	Roberts and DiFiglia (1988), Emerich et al. (1991), Pundt et al. (1996)	+	?
SINs: NKX2.1,		?	+ Aubry et al. (2008), El-Akabawy et al. (2011)
SINs: Mash1,		?	+ El-Akabawy et al. (2011)
SINs: Dlx1, Dlx2		?	+ El-Akabawy et al. (2011)
VMAT1		?	+ Kallur et al. (2006)
Striatal enriched phosphoprotein, STEP	Fricker et al. (1994)	+ *[in P zones]*	
**Efferent projections**
Direct pathway : MSNs > GPi	Wictorin et al. (1990b)	+	?
Direct pathway : MSNs > SNr	Wictorin et al. (1989, 1990b)	+	Ma et al. (2012)
Indirect pathway : MSNs > GPe	Wictorin et al. (1989, 1990b), Clarke and Dunnett (1993), Olsson et al. (1995)	+ *[from P zones]*	Nasonkin et al. (2009)
[outgrowth into neocortex]	Wictorin et al. (1990a)	+ *[aberrant]*
**Afferent projections**
Neocortex layer III and V, glutamate, topographic	Pritzel et al. (1986), Wictorin and Björklund (1989), Wictorin et al. (1989)	+ *[P and NP zones]*	Ma et al. (2012)
Substantia nigra compacta, dopamine (CCK-)	Pritzel et al. (1986), Clarke et al. (1988), Wictorin et al. (1989), Labandeira-García et al. (1991), Clarke and Dunnett (1993), Capetian et al. (2009)	+ *[into P zones]*	Ma et al. (2012), Arber et al. (in press)
Raphé nucleus, serotonin	Wictorin et al. (1988), Labandeira-García et al. (1991), Pierret et al. (1998), Petit et al. (2002)	+	?
Thalamus, VA, VL …, glutamate (?)	Pritzel et al. (1986), Wictorin et al. (1988)	+	?
**Electrophysiology**
*in vitro* membrane properties	Walsh et al. (1988), Siviy et al. (1993)	+ *[normal or aberrant]*	?
Local connections > EPSPs	Walsh et al. (1988)	+ *[EPSPs + IPSPs]*	?
Patch-clamp features of inward rectifying current	Surmeier et al. (1992)	+ *[relatively normal]*	?
responses to pharmacological challenges	Nakao et al. (2000), Chen et al. (2002)	+	?
Monosynaptic EPSPs cortex > MSN	Rutherford et al. (1987), Walsh et al. (1988), Xu et al. (1991)	+ *[aberrant]*	?
Monosynaptic EPSPs nigra > MSN		?	?
Monosynaptic EPSPs thalamus > MSN	Xu et al. (1991)	+ *[aberrant]*	?
Monosynaptic IPSPs : MSN > GPe		?	?
Monosynaptic IPSPs : MSN > GPi	Nakao et al. (1999)	+	?
Monosynaptic IPSPs : MSN > SNr		+ *[aberrant]*	?
LTP and LTD plasticity at corticostriatal synapse	Mazzocchi-Jones et al. (2009, 2011)	+	?
Fast spiking interneurons		?	?
**Receptors**
dopamine D1, D2 receptors	Isacson et al. (1987), Deckel et al. (1988b), Liu et al. (1990), Lu and Norman (1993)	+	?
glutamate NMDA receptors	Siviy et al. (1993), Hussain et al. (2004)	+	± Nasonkin et al. (2009)
GABA receptors		?	?
ACh muscarinic receptors	Isacson et al. (1987), Deckel et al. (1988a), Liu et al. (1990), Lu and Norman (1993)	+	?
µ-Opiate receptors	Isacson et al. (1987), Lu and Norman (1993)	+ *[in P zones]*	?
cannabinoid CB1 and CB2 receptors		?	?
adenosine receptors		?	?
CCK receptors	Lu and Norman (1993)	+	?
**Neurochemistry**
Striatal derived GABA release in striatum or GP	Sirinathsinghji et al. (1988), Campbell et al. (1993)	+	?
Striatal CCK regulation of DA release	Sirinathsinghji et al. (1993)	+	?
**Functional recovery**
Motor—activity and locomotion	Isacson et al. (1984), Deckel et al. (1986)	+	Ma et al. (2012)
Motor asymmetry, rotation	Dunnett et al. (1988), Norman et al. (1988, 1989)	+	+Song et al. (2007), Delli Carri et al. (2013)
Motor coordination and balance (e.g., rotarod)	Giordano et al. (1990)	+	–El-Akabawy et al. (2012) + Lee et al. (2006), Ma et al. (2012)
Motor skills—e.g., paw reaching	Dunnett et al. (1988), Montoya et al. (1990), Döbrössy and Dunnett (2001), Klein et al. (2013)	+	?
Sensorimotor, e.g., neglect	Deckel et al. (1986)	+	?
Motor learning	Mayer et al. (1992), Döbrössy and Dunnett (1998), Brasted et al. (1999)	+	?
Cognition—classic prefrontal tasks, e.g., delayed alternation	Deckel et al. (1986), Dunnett and White (2006)	+	?
Cognition—simple learning tasks, e.g., passive avoidance	Piña et al. (1994a), Giordano et al. (1998)	+	?
Cognition—S-R vs. incentive based learning		?	?
Cognition—executive function, e.g., set shifting		?	?
Psychiatric—impulsivity and disinhibition	Reading and Dunnett (1995)	+	?
Psychiatric—sensitivity to reward and motivation		?	?

*^†^This table provides a summary of the range of morphological, neurotransmitter, receptor, molecular, cellular, functional features of the normal striatum against which primary fetal and stem cell derived grafts have been evaluated. Comprehensive review is beyond the scope of the present application; see individual references for details*.

*^††^See Table 1 for full description*.

*Abbreviations: CCK, cholecystokinin; CR, calretinin; Dyn, dynorphin; Enk, enkephalin; GABA, gamma-amino butyric acid; GCN, giant cholinergic interneurons; GPe, external segment of globus pallidus; GPi, internal segment of globus pallidus; LTD, long-term depression; LTP, long-term potentiation; MSN, striatal medium spiny projection neurons; NOS, nitric oxide synthase; NPY, neuropeptide Y; PSC, pluripotent stem cell; PV, parvalbumin; SIN, striatal interneuron; SNc, substantia nigra pars compacta; SNr, substantia nigra pars reticulata; SOM, somatostatin; SP, substance P; VA, ventral anterior nucleus ; VL, ventrolateral nucleus*.

^*^Notes. + Present. – Absent, ? no known published data.

## Normal striatal development

The mammalian striatum develops within the ventral telencephalon; specifically from the whole ganglionic eminence (WGE), which can be further subdivided into the lateral, medial and caudal segments (LGE, MGE and CGE, respectively). Striatal projection neurons originate predominantly in the LGE whereas striatal interneurons are born primarily in the MGE (Evans et al., 2012). During striatal development, subsets of marker genes (transcription factors, etc.) can be used to differentiate between different neuronal types, and between different stages of development. Consequently, the characterization of the expression of key genes during normal striatal development has become an essential guide for developing and validating protocols for *ex vivo* differentiation of PSCs to similar fates.

For rat and mouse PFC allografts, it has been determined empirically that the optimal stage of fetal development for tissue donation coincides with the peak of neurogenesis of the relevant target population, i.e., around embryonic day E14-15 for striatal grafts (Dunnett and Björklund, 2000). By contrast, the donor age for human or primate xenograft or allograft studies is typically determined by selecting embryos at the equivalent Carnegie stage known to be effective from rodent studies, i.e., 7–8 weeks of gestation (9–10 weeks post last menstruation; 17–28 mm crown-rump length) for human striatal tissue (Butler and Juurlink, 1987; O’Rahilly and Müller, 1987; Dunnett and Björklund, 2000). There is as yet insufficient data from different clinical studies, with inadequate information on either donor age or functional outcomes, to establish the validity of this essentially empirical principle, not least because of the multiplicity of other factors that also contribute to graft viability and function (Freeman et al., 2011).

Within the adult striatum, neurons are heterogeneous and can be subdivided according to size, density of spines, and utility of neurotransmitters and neuropeptides. Striatal MSNs constitute 90–95% of all striatal neurons in rodents (and about 80–85% primates) and are the main output projection neurons of the striatum (Gerfen, 1992). The MSNs utilize the inhibitory gamma-amino butyric acid (GABA) as their principle neurotransmitter (Gerfen, 1992; Feng et al., 2014) and subpopulations also use enkephalin, dynorphin and/or substance P as co-transmitters (see Table 2). They also stain for DARPP-32 which, as mentioned earlier provides a commonly-used and convenient marker of the MSN cell population. The remaining neurons are spiny and aspiny interneurons (5–10% rodents and up to 20% in primates) which are classically subdivided into 4 types: parvalbumin-positive, calretinin-positive, neuropeptide Y, somatostatin and neuronal nitric oxide synthase (NPY/SS/nNOS) positive, all of which are GABAergic and of medium size, and the giant aspiny cholinergic (choline acetyltransferase, ChAT-positive) interneurons ((Freeman et al., 1995; Durieux et al., 2011; Feng et al., 2014), see Table 2). The functional contribution of these interneuronal subpopulations to striatal processing is not well characterized, although there has been an increase in research in this area recently (Tepper et al., 2010). It is clear, however, that the striatal MSNs do not simply relay untransformed information from cortex to globus pallidus, but information throughout is modulated in the course of the striatal contribution to action selection, motor learning and habit formation. Thus, it is highly likely that the striatal cholinergic and GABAergic interneurons play key roles in effecting these functional processes (Do et al., 2012). To take just one example, Calabresi and colleagues show that the nitric oxide and cholinergic interneurons exert feed-forward control of the excitability of the MSNs to coordinate alternative forms of neuroplasticity during motor learning (Centonze et al., 1999). In addition, aberrant GABAergic cortical interneuron development is associated with some neuropsychological disorders such as schizophrenia (Arber et al., in press).

A further level of organization is important for normal striatal function: the parallel direct and indirect pathways for downstream information transfer. One subpopulation of striatal MSNs projects directly to their principle downstream targets via the internal segment of the globus pallidus and substantia nigra pars reticulata, and thence to thalamus and brainstem. A second subpopulation projects to the external segment of the globus pallidus which interacts reciprocally with subthalamic nucleus prior to then converging via this indirect route, on the same downstream targets. Whereas the striatal matrix contributes to both direct and indirect pathway projections, there is growing evidence that the striosomes contribute to the direct pathway only (Fujiyama et al., 2011). Moreover, the direct and indirect pathways differ in their co-transmitters which they operate with GABA (substance P and dynorphin vs. enkephalin, respectively (Gerfen and Young, 1988; Albin et al., 1989)), and in the subtypes of dopamine receptors to which they differentially respond (excitatory D1 vs. inhibitory D2 receptors respectively (Gerfen and Young, 1988; Jimhénez-Castellanos and Graybiel, 1989; Gerfen et al., 1990)). The importance of this organization to striatal repair lies in the highly influential model introduced by DeLong (1990), based on initial distinctions introduced by Albin et al. (1989), which suggests that different hyperkinetic vs. hypokinetic motor disorders can be characterized in terms of imbalances between direct and indirect pathways outputs. Thus, so-called “hyperkinetic” conditions including HD result from excess activation of the indirect pathways, as evidenced for example by preferential loss of enkephalinergic over substance P MSNs in a *post mortem* HD brain (Reiner et al., 1988), and which may be corrected symptomatically by D2 down-regulation. The task for any reparative therapy—whether based on cell replacement or some other method of inducing intrinsic reorganization and plasticity—is to restore the balance between counterbalanced net excitatory and inhibitory pathways and their connections, not simply restoring striatal input-output relays. The organization of the neostriatum in health and disease is fundamental to its normal function and dysfunction, and sets precise challenges of how to restore a sufficient match to the healthy organization if any reparative therapy is to be effective.

## Striatal grafting

The most widely used models for studies of striatal repair and transplantation have been excitotoxic lesions of the striatum in rats and primates. Although transgenic models have recently become more widely available, the cellular pathology is substantially more widespread throughout the brain and body than seen in the human condition (Morton et al., 2000). As a consequence, notwithstanding their utility for developing other strategies for neuroprotection, genetic mutation models have proved to be less suitable for studying cell-based repair of focal striatal degeneration (Dunnett et al., 1998) than the classical approach using excitotoxins. Of these, the quinolinic acid (QA) lesion model of HD is the preferred contemporary option (Beal et al., 1986), as it depletes the MSNs relatively selectively and without the additional side effects of earlier alternatives, such as kainic or ibotenic acids. Recently, in addition to inducing MSN cell death, it has also been shown that QA causes a decrease in the number of parvalbumin and NPY interneurons, sparing calbindin and ChAT positive interneurons, similar to the selective profile of cell loss in human HD (Feng et al., 2014).

Striatal transplantation is a relatively straightforward procedure involving injection of striatal primordium, typically derived from the developing fetal forebrain and prepared as a dissociated cell suspension, directly into the host striatum under stereotactic guidance (Schmidt et al., 1981; Isacson et al., 1984). Similar protocols have been widely used in many labs worldwide and found to be relatively simple, reliable and reproducible, and rat allograft studies dominate the striatal-grafting field. Such experiments have allowed us to understand what constitutes an optimal striatal graft, not only in terms of cell survival, differentiation and anatomical integration, but also in terms of functional impact on the host brain and host behavior (Dunnett et al., 2000) (see Table 2).

Striatal mouse allograft experiments also exist but as yet have not been as forthcoming due to inconsistencies in the long term survival of grafts in the mouse brain (Roberton et al., 2013). Furthermore, human-rat xenograft experiments have served not only as a precursor to clinical trials of cell transplantation in man, but also as an important model to study normal human fetal development and cell fate. Striatal allografts in primates have been used both for scaling up protocols and to demonstrate similar profiles of functional motor and cognitive recovery in the more complex primate basal ganglia (Kendall et al., 1998; Palfi et al., 1998), prior to clinical application. Finally we now have *in vivo* and *post mortem* evidence accumulating from human-human allografts in HD patients that have entered the first generation of clinical trials of PFC transplantation in this disease (Bachoud-Lévi et al., 2000; Freeman et al., 2000; Rosser et al., 2002; Cicchetti et al., 2011).

The comparison between within- and cross-species studies highlights the fact that graft growth and recovery of function must be considered within the context of species-specific rate of neuronal development of the donor tissue, which translates directly into very different timelines for experimental studies of tissue derived from mice, rats, primates and man, irrespective of the host. Collectively, PFC transplant studies have utilized a range of cell preparation methods and have reported a range of outcome measures including cell morphology, neurotransmitter type, molecular markers, projections, electrophysiology, and functional recovery; so what have such experiments highlighted and how should this be applied to the characterization and evaluation of PSC-derived donor cells?

## Cell morphology

Rat WGE grafted into the QA-lesioned host striatum reveals a relatively homogenous population of neuronal cell bodies within the graft as indicated by general histological stains such as NEUN or cresyl violet. However, from the earliest grafting experiments it is clear that different neuronal types develop within the striatal tissue grafted. For example Golgi experiments highlighted the presence of at least 5 types of medium sized, spiny neurons as well as aspiny neurons (Helm et al., 1990), and non-striatal (e.g., pyramidal) as well as striatal cell types (Clarke et al., 1994). Early use of neurochemical stains revealed two morphologically distinct regions, acetylcholinesterase (AChE positive) “P-zones” (30–40%) and AChE negative non-P (NP)- zones (Isacson et al., 1987; Graybiel et al., 1989; Wictorin et al., 1989; Pakzaban et al., 1993), with the latter having at least three fold less DARPP-32 positive MSNs (Wictorin and Björklund, 1989; Nakao et al., 1994). In addition to DARPP-32 expression, the P-zones positively stain for striatal interneurons whereas the NP zones are comprised primarily of non-striatal cell types that also originate from within the ganglionic eminence primordium, such as pallidal and cortical neurons (Clarke et al., 1994). These distinctive zones have been shown in rat (Watts et al., 2000a,c), human (Cisbani et al., 2013) and to a lesser degree in mouse allografts (Döbrössy et al., 2011) and human-rat xenografts (Grasbon-Frodl and Brundin, 1997; Grasbon-Frodl et al., 1997; Sanberg et al., 1997), showing that such autonomous patterning is not species-specific. Although when first observed it was natural to hypothesize that the grafts were developing an intrinsic striosome-matrix organization (Isacson et al., 1987), it was quickly realized that this was not the case. Rather, the ganglionic eminence gives rise to diverse populations of cortical, pallidal and other deep forebrain progenitors as well as the target precursors of striatal fate(s). Thus, the patches of positive staining for AChE co-localized with a broad range of markers of all striatal types, striosomes as well as matrix, that aggregated into a patchy organization (designated “P zones” to distinguish the term from Gerfen’s “patch” terminology for striosomes) interspersed with a non-P compartment (or “NP-zones”) comprising neurons of primarily cortical, pallidal and other non-striatal phenotypes (Graybiel et al., 1989).

To date PSC-based protocols have not clearly identified a similar differentiation into different zones within the grafts (see Table 2). This could suggest a relative purity of the differentiated PSC-derived neurons (e.g., relatively pure yields of striatal MSNs), with no contamination from cortical and other forebrain phenotypes, exactly as designed. Alternatively, the grafted PSC-derived neurons typically show continuing expression of markers of immaturity, such as nestin and doublecortin, and it may simply be that the markers necessary to reveal a mature heterogeneity of organization are not yet expressed at the relatively short survival times so far studied. Thirdly, the intercellular signals that guide self-organization and aggregation of striatal-like cells in PFC grafts may simply not be expressed in PSC-derived neurons within the grafts. Crucial to interpretation of this first obvious difference in histological descriptions of PSC-derived vs. PFC-derived striatal transplants, lies the question whether the distinct P-zones seen in classical striatal graft studies are even required (let alone optimal) for a functional graft, or do they only serve as a way of demonstrating that conventional grafts are intrinsically suboptimal by virtue of contamination with irrelevant cell types.

When primary grafts involve a fetal dissection that is restricted to LGE (the zone within which the bulk of the MSNs originate), the size of the P-zones increases to 80–90% of the total graft volume, and the proportion of DARPP-32 expression and MSN-type neurons within the grafts is correspondingly increased (Pakzaban et al., 1993; Olsson et al., 1995). Moreover, LGE-restricted grafts showed more selective axonal growth towards the GPe when compared to MGE grafts (Olsson et al., 1995). It has therefore been argued that clinical grafts should be based on a restricted LGE dissection in order to maximize the number of MSNs within the grafts and minimize contamination by non-striatal cells (Brundin et al., 1996). However, although the proportion of DARPP-32 neurons is increased in LGE grafts, not only the total graft volume but also the volume of the patches is greater in WGE grafts (Watts et al., 2000b). Moreover, there is clear evidence of a more extensive functional recovery with WGE than LGE grafting. Together, these observations suggest that the reconstitution of all striatal cell types—both MSNs and interneurons—within the graft tissue is important in optimizing graft function (Dunnett, 2000). Interestingly, a recent experiment in which LGE cells from a GDNF^−/–^ or GDNF^+/+^ mouse were grafted into the ventricles of WT mice has revealed that grafts devoid of GDNF-expressing cells appeared smaller at 2 week survival, and over time had less DARPP-32 and TH innervation than grafts of wild-type tissue (Chermenina et al., 2014). In the adult striatum GDNF is produced by the GABAergic and cholinergic interneurons (Bizon et al., 1999; Hidalgo-Figueroa et al., 2012), again suggesting that proper maturation of the major populations of projection neurons within striatal grafts is critically dependent upon factors only produced by inclusion of striatal interneurons.

The use of human donor cells has produced slightly different results to rat allograft experiments. At most only 30% of the human LGE tissue grafted into the QA-lesioned rat striatum consisted of P-zones (Grasbon-Frodl et al., 1996), although this could be put down to species-specific rate of maturation (Tyson and Anderson, 2013). However, a later experiment did show a decrease in the number of apomorphine induced rotations compared to post-lesion tests despite histology showing no definitive P-zones (Sanberg et al., 1997) suggesting that the function of the graft is not solely dependent on the specific striatal cellular markers but depends on how well the graft connects with its host.

Taken together, these observations suggest that if we are to maximize functional impact we need to move beyond the restitution of lost MSNs in a PSC-derived graft; we need to both understand and pay attention to the full complement of striatal and non-striatal cell types, along with their internal organization, established within the grafted tissue and design graft composition accordingly. Despite the weight of evidence indicating WGE grafts being favorable over the LGE, and increased P-zones being preferred for functionality, current protocols directing PSC to striatal fates largely focus on producing and testing an MSN-pure, LGE-like phenotype, and with rare exceptions are less concerned with the detailed cell morphology of the graft. Upon histology, emphasis is placed upon molecular makers or neurotransmitter makers indicative of an MSN, and of course whether there are any proliferating cells remaining to ensure no possibility of tumor formation. No histological studies have yet looked in depth at spine density or the numbers of interneurons, and only one published protocol has assessed the presence of a single MGE marker (Nkx2.1) in grafts (Ma et al., 2012). The differentiation strategies typically adopted follow guidelines from fetal development to apply a series of transcription factors and other switches to progressively rostralise and dorsalise/ventralise fates of differentiating cells converging on a particular striatal population. This does not preclude the development of interneurons as well as MSNs within the grafts as appropriate to WGE (rather than pure LGE) targets. Rather, the selective focus on the MSN target means that insufficient attention is paid to the switch signals and markers of intermediates in cell preparation and final neuronal fates of the differentiated cells within PSC-derived grafts to allow us to determine the extent to which complex striatal phenotypes are already being achieved at present. Moreover, we need also to consider whether alternative differentiation protocols might yield more comprehensive fate outcomes than the pure MSN composition of the grafts *per se*.

## Circuit formation

It is important that striatal grafts interact with the host environment, i.e., that afferent and efferent connections are made. Immunohistochemical staining can easily identify DA input to the grafts (by TH staining of host-derived DA axons and terminals), but for a more thorough analysis species-specific neurofilament antibodies or optogenetic labels may need to be used to allow more accurate visualization and quantification of retrograde and anterograde fiber outgrowth (Wictorin et al., 1990a, 1991). Typically, inputs and outputs from PFC striatal grafts originate selectively from the P-zones (Wictorin et al., 1989), i.e., the compartment comprising the subpopulation of striatal-like neurons within the grafts. Moreover, combined Golgi staining, immunohistochemical labeling, and anterograde and retrograde pathway tracing at the electron microscopy level has shown that host cortical inputs establish morphologically appropriate symmetric synapses on the heads of the spines of grafted MSN output neurons projecting to the GP, whereas the host DA inputs make asymmetric synapses on the necks of spines of the same neurons (Clarke et al., 1988; Clarke and Dunnett, 1993), clearly demonstrating the key elements of authentic circuit reconstruction. Similarly, both electrophysiological (Rutherford et al., 1987; Siviy et al., 1993) and neurochemical (Sirinathsinghji et al., 1988; Campbell et al., 1993) studies indicate a clear relay of afferent and efferent information between neurons within the grafts and host circuits. However, how the striatal interneurons are contributing to grafts is still unknown (or, at least, nowhere demonstrated directly) other than by inference from our understanding of normal circuit function.

Again, although several PSC-based protocols have checked by whole-patch clamp analysis that the cells within the graft express action potentials characteristic of neurons (Delli Carri et al., 2013), only one has looked closely at projections within the graft. Specifically Ma and colleagues showed that there was host cortical and nigral inputs to the grafts and that there were efferent projections afforded to the SN from the grafts (Ma et al., 2012). It is likely that as protocols become more advanced, assessing input and output connections and neurotransmitter release will be routine as is the case for primary grafts.

## Functional recovery

Although striatal grafts can stain with striatal markers and show connections to appropriate areas, whether these grafts have successfully restored striatal circulatory and neurotransmitter levels can only be determined by looking at functional recovery, the ultimate goal of cell replacement therapy. There is a rich literature on studies demonstrating significant functional recovery following PFC striatal transplantation on a broad range of motor and cognitive tests in unilateral and bilateral striatal lesioned rats (reviewed in Dunnett et al., 2000). These include recovery in tests of motor function including locomotor activity, rotation and rotational asymmetry, coordination and balance, and skilled reaching and manipulation skills, and in the cognitive domain including recovery in active and passive avoidance, active and passive avoidance learning, spatial navigation and rule learning in T and water mazes, and operant learning and delayed response tasks. Importantly, these tests reveal clear recovery on tasks dependent upon intact cortical fronto-striatal systems, clearly implicating the restoration of circuit connectivity in functional recovery (Dunnett et al., 2000). Most importantly, the use of transfer of learning experiments in a lateralized operant habit learning task has clearly indicated that the grafted tissues provide a necessary substrate for the formation of new learning (rather than simply being necessary for the execution of the response) (Brasted et al., 1999), and direct intracellular and extracellular recording in striatal slices indicates the restoration of characteristic synaptic plasticity at the reformed corticostriatal synapse between host circuits and graft cells necessary to mediate such new learning (Mazzocchi-Jones et al., 2009). These analyses indicate that it is not sufficient simply for a striatal graft to replace lost neurons of the appropriate type and in their appropriate location; rather, they must integrate into the host circuitry to restore striatal modulation of cortical and thalamic information in the flexible selection of action based on experience and the history of reinforcement. Moreover, once the substrate is restored, the grafted animal, and presumably also the patient, requires to restore the lifetime of acquired motor skills and habits lost to the disease process (Döbrössy and Dunnett, 2001).

## The challenges for pluripotent stem cell grafts

Up to this stage in the commentary, we have established that striatal grafts derived from implants of the developing striatal primordium in the fetal ganglionic eminence can survive transplantation, differentiate into appropriate populations and subpopulations recapitulating the normal striatal phenotype, connect and integrate with the host brain making appropriate synaptic contacts to restore the basic components of the striatal circuitry, and function according to neurochemical, electrophysiological and behavioral criteria sufficient to alleviate a range of deficits in both motor and cognitive domains associated with striatal neuronal cell loss and damage. Moreover, the observed recovery is in processes such as S-R habit learning known to be dependent upon restitution of functional cortical-striatal-pallidal circuits, mediated by intracellular and synaptic plasticity involving LTP and LTD, which are in turn dependent upon complex synaptic and intracellular signaling processes, integrating signals of reward mediated by afferent dopaminergic and other brainstem and thalamic inputs, and modulated by appropriate interaction with interneurons within the local striatal networks. On the one hand this sets the range of functional assessments that are required to establish the functional viability and to characterize the extent of functional recovery offered by an alternative cell source, such as is provided by PSCs. On the other hand, given the complexity of striatal organization, and the demonstrated ability of authentic striatal primordium developing within PFC grafts, it is to us implausible that relatively pure populations of MSNs will have the capacity to either integrate or function to a similar degree. This is not in any way to suggest that the MSNs are not necessary or indeed fundamental. Rather, the known principles of normal striatal organization and development demand restoration of a functional complexity that is greater than initially envisaged.

The existing protocols based on PSC transplantation, although restricted in their scope, already provide good evidence of the capacity of such grafts to provide a replacement of new neurons that survive in the striatum, express multiple markers of MSN differentiation, in particular GABAergic phenotypes and DARPP32-positivity, limited outgrowth of graft neurites into the host brain and re-afferentation by at least host dopamine fibers, and partial recovery on some simple tests of motor asymmetry (see Table 2). Anatomical and electrophysiological characterization of graft cells’ integration into host neuronal circuitries, and recovery on a broader and more comprehensive profile of functional tests has largely not yet been studied, but less functional efficacy than is provided by existing PFC-derived grafts would not be unexpected. This is unsurprising, based on the so far selective targeting of one specific cell type, the MSN projection neuron; in the rare cases where a broader characterization of interneuronal markers has been sought they have not been identified. We can identify the need to progress the field at three levels.

First, a broader characterization of PSC-based grafts is required, in terms of cellular composition, molecular differentiation of multiple striatal phenotypes, electrophysiological and anatomical connectivity, and functional efficacy in more complex motor and cognitive functions than the simple motor screening tests used to date. Such a broad profile of characterization should become the norm rather than the exception.

Second, the strategies for PSC differentiation to achieve a cellular composition in the grafts that better reflects the full composition of striatal interneurons, projection neurons and glia. As we understand more about striatal development and can better control distinct striatal fates, it is plausible to design additive experiments to determine the combinations of cell types of which the grafts are composed, and the components of circuit reconstruction that are necessary and sufficient to achieve different degrees and profiles of recovery in distinct motor, motivational, cognitive and behavioral functional domains.

Thirdly, while PSC-derived grafts fall short of functional efficacy achieved by PFC striatal tissues, the latter remains the “gold standard”. However, PFC grafts are themselves not optimal; they can vary markedly from case to case, the precise composition of cell types, internal connections and rewiring and complete functional recovery, never achieves the total repair that restores the damaged system fully to normal. Especially with the much greater degree of control we can potentially apply to PSC-derived protocols, our goal should not simply be to provide a more practical alternative that can match the present PFC standard, but a new generation of cell therapy that can substantially supersede our present still poor levels of competence.

The advantage that was offered by PFC -derived tissues in the initial development of cell transplantation for HD lies in the capacity of fetal neurons developing *in situ* for growth, integration and function; that is what they have evolved to do, and are features that are retained by the cells when transplanted into an adult damaged or diseased brain environment. The challenge for the stem cell biologist is to understand that process and design a more practical and effective replacement that can still achieve the same complex fate, both in terms of intrinsic capacity and of their ability to respond to the signals provided by the host environment. The preceding consideration of PFC-derived striatal grafts sets a high baseline of necessary functional analysis for future generations of PSC-derived cell therapy, not just as an empirical measure of functional efficacy and recovery, but to determine and understand the mechanisms of recovery and provide a rational foundation for the design and implementation of an effective replacement cells.

## Conflict of interest statement

The authors declare that the research was conducted in the absence of any commercial or financial relationships that could be construed as a potential conflict of interest.
